# Continuous 7-Month Internet of Things–Based Monitoring of Health Parameters of Pregnant and Postpartum Women: Prospective Observational Feasibility Study

**DOI:** 10.2196/12417

**Published:** 2020-07-24

**Authors:** Johanna Saarikko, Hannakaisa Niela-Vilen, Eeva Ekholm, Lotta Hamari, Iman Azimi, Pasi Liljeberg, Amir M Rahmani, Eliisa Löyttyniemi, Anna Axelin

**Affiliations:** 1 Department of Nursing Science University of Turku Turku Finland; 2 Department of Obstetrics and Gynecology Turku University Hospital Turku Finland; 3 Faculty of Medicine University of Turku Turku Finland; 4 Faculty of Communication Sciences University of Tampere Tampere Finland; 5 Department of Future Technologies University of Turku Turku Finland; 6 School of Nursing and Department of Computer Science University of California Irvine, CA United States; 7 Department of Biostatistics University of Turku Turku Finland

**Keywords:** prenatal care, postnatal care, wearable electronics, biosensing, cloud computing, mHealth, physical activity, sleep, heart rate

## Abstract

**Background:**

Monitoring during pregnancy is vital to ensure the mother’s and infant’s health. Remote continuous monitoring provides health care professionals with significant opportunities to observe health-related parameters in their patients and to detect any pathological signs at an early stage of pregnancy, and may thus partially replace traditional appointments.

**Objective:**

This study aimed to evaluate the feasibility of continuously monitoring the health parameters (physical activity, sleep, and heart rate) of nulliparous women throughout pregnancy and until 1 month postpartum, with a smart wristband and an Internet of Things (IoT)–based monitoring system.

**Methods:**

This prospective observational feasibility study used a convenience sample of 20 nulliparous women from the Hospital District of Southwest Finland. Continuous monitoring of physical activity/step counts, sleep, and heart rate was performed with a smart wristband for 24 hours a day, 7 days a week over 7 months (6 months during pregnancy and 1 month postpartum). The smart wristband was connected to a cloud server. The total number of possible monitoring days during pregnancy weeks 13 to 42 was 203 days and 28 days in the postpartum period.

**Results:**

Valid physical activity data were available for a median of 144 (range 13-188) days (75% of possible monitoring days), and valid sleep data were available for a median of 137 (range 0-184) days (72% of possible monitoring days) per participant during pregnancy. During the postpartum period, a median of 15 (range 0-25) days (54% of possible monitoring days) of valid physical activity data and 16 (range 0-27) days (57% of possible monitoring days) of valid sleep data were available. Physical activity decreased from the second trimester to the third trimester by a mean of 1793 (95% CI 1039-2548) steps per day (*P*<.001). The decrease continued by a mean of 1339 (95% CI 474-2205) steps to the postpartum period (*P*=.004). Sleep during pregnancy also decreased from the second trimester to the third trimester by a mean of 20 minutes (95% CI –0.7 to 42 minutes; *P*=.06) and sleep time shortened an additional 1 hour (95% CI 39 minutes to 1.5 hours) after delivery (*P*<.001). The mean resting heart rate increased toward the third trimester and returned to the early pregnancy level during the postpartum period.

**Conclusions:**

The smart wristband with IoT technology was a feasible system for collecting representative data on continuous variables of health parameters during pregnancy. Continuous monitoring provides real-time information between scheduled appointments and thus may help target and tailor pregnancy follow-up.

## Introduction

Monitoring and follow-ups during pregnancy are vital to ensure the health and well-being of pregnant women and their unborn infants. Thus far, monitoring of pregnancy is performed at scheduled appointments by health care professionals in maternity care units [1]. Instead of intermittent measurements tied to time and place, remote and continuous monitoring could provide significant opportunities for health care professionals to observe the health-related parameters of their patients [2] and detect abnormal changes in maternal adaptation to pregnancy and liability to pregnancy complications early. Remote monitoring might support personalized care as maternity care could be tailored according to the received information. A personalized monitoring approach could also enhance a woman’s self-management because it makes the woman more aware of her health and might commit her to pregnancy care [3].

The Internet of Things (IoT) provides methods for ubiquitous and continuous maternity monitoring. The IoT is a high-level network of objects (ie, things) that are wirelessly connected to servers to provide efficient and comprehensive services [4,5]. In practice, the pregnant woman wears sensors that monitor health-related parameters, enabling her and health care professionals to track the data through web-based user interfaces anywhere and at any time [5]. Recently, the use of various eHealth applications in pregnancy care has sharply increased—for example, for remote monitoring of blood glucose—and the number of maternity care visits for women with gestational diabetes mellitus has decreased with no differences in maternal or neonatal outcomes noted [6,7]. Further, pulse and blood pressure sensors in a smartphone application have been developed, and the feedback from pregnant women has been positive [8,9].

Physical activity and sleep are significant for a pregnant woman’s general well-being and quality of life [10]. Physical activity decreases as pregnancy progresses [11], and sleep disorders are common among pregnant women, especially during the third trimester and during the postpartum period [12]. Both physical activity and sleep are usually measured by subjective self-reports [11-13], which is complicated because self-reports may over or underestimate the duration of sleep or activity [13,14]. On the other hand, device-based monitoring has the potential to increase physical activity because having visibility to one’s daily activity is known to be an incentive to increase physical activity [15]. Although smart wristbands are widely utilized to measure activity, sleep, and heart rate, their use in pregnant women is still scarce [2]. Continuous device-based monitoring would provide unique and representative data on the levels and changes of activity and sleep during pregnancy and the postpartum period. Furthermore, heart rate measures could be utilized to evaluate the intensity of the physical activity of pregnant and postpartum women. Continuous monitoring could also be useful to target interventions to those needing them the most and to measure health outcomes systematically.

This study aimed to evaluate the feasibility of continuous monitoring of health parameters (physical activity, sleep, and heart rate) of nulliparous women throughout pregnancy and until one month postpartum with a smart wristband and an IoT-based monitoring system.

## Methods

### Study Design and Settings

**Figure 1:**
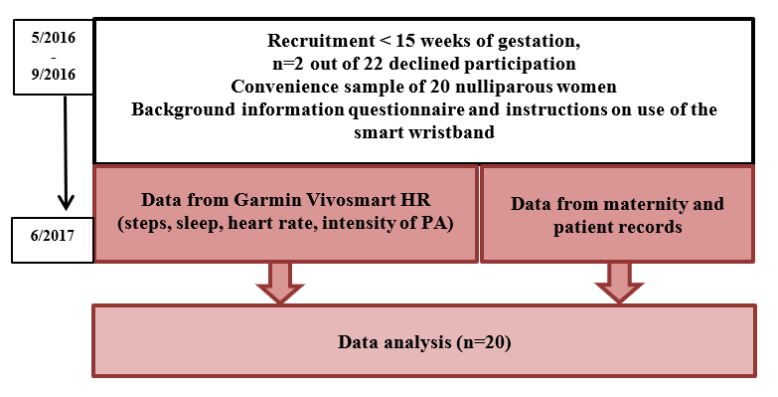
The study design.

This study was conducted as a prospective observational feasibility study on nulliparous women attending two maternity outpatient clinics in Southern Finland between May 2016 and June 2017. Physical activity, sleep, and heart rate data were collected with a smart wristband integrating a photoplethysmogram bio-sensor to measure heart rate [16] and an inertial measurement unit to track activity and sleep [17]. The study design is presented in Figure 1.

The study was carried out per the Code of Ethics of the World Medical Association (Declaration of Helsinki) and approved by the Joint Ethics Committee of the Hospital District of Southwest Finland (35/1801/2016) and the university hospital. Permission to use Garmin Vivosmart (HR, Garmin) smart wristbands in this study was obtained from the manufacturer. The study was not registered due to the feasibility design.

### Participants and Recruitment

In Finland, all pregnant women are offered a screening ultrasound free of charge at the end of their first trimester. A convenience sample of 20 pregnant women was recruited from this visit. Criteria for eligibility were women (1) expecting their first child, (2) ≥18 years of age, and (3) ≤15 of weeks of singleton gestation. Women were excluded if they did not understand Finnish or English or did not have a PC, tablet, or mobile device with which to synchronize data.

The midwives at the maternity outpatient clinics informed the eligible women about the study. After providing oral and written information, the midwives asked permission for the researchers to be in contact with potential participants (N=22). The researchers explained the study purpose and procedures to the women by telephone and scheduled a meeting if a woman was willing to participate. Two women declined participation because they felt that they would not wear the smart wristband. Written informed consent was obtained from all participants at the meeting with the researchers. In addition, a smart wristband and instructions on how to use it were given to each participant, and background information was obtained through a questionnaire.

### Outcome Measures and Data Collection

Maternal background characteristics of age, BMI, marital status, education, employment status, smoking, and pre-pregnancy physical activity habits were collected with a questionnaire at the meeting with the researcher. Data on pregnancy and delivery were collected from the maternity card and hospital electronic patient records.

Physical activity, sleep, and heart rate data were collected objectively using a Garmin Vivosmart, small (21 mm × 12.3 mm) and light (29.6 g) smart wristband. The device has shown an acceptable level of validity for step counts tested under laboratory conditions against the Optogait system (OPTOGait, Microgate Srl) and a manual hand counter on the treadmill [18,19]. The device detected sleep automatically based on heart rate and hand movements during regular sleep hours, not during the day. Physical activity intensities were estimated by comparing heart rate data with the data collected by an accelerometer sensor when acceleration was detected, in comparison to the patient’s average resting heart rate. If the heart rate sensor was not on, the device calculated the number of steps per minute to evaluate moderate-to-vigorous physical activity (MVPA) minutes. At least 10 consecutive minutes must be recorded to earn intensity minutes so that either step count rate or heart rate was elevated above the predefined moderate-intensity threshold [20]. The women were instructed to wear the activity tracker for 24 hours a day during their pregnancy and for 1 month after delivery, totaling 7 months. They were advised to synchronize the devices once a day or at least when charging the devices every 5 days. Data were accessible for both the researchers and the women.

**Figure 2:**
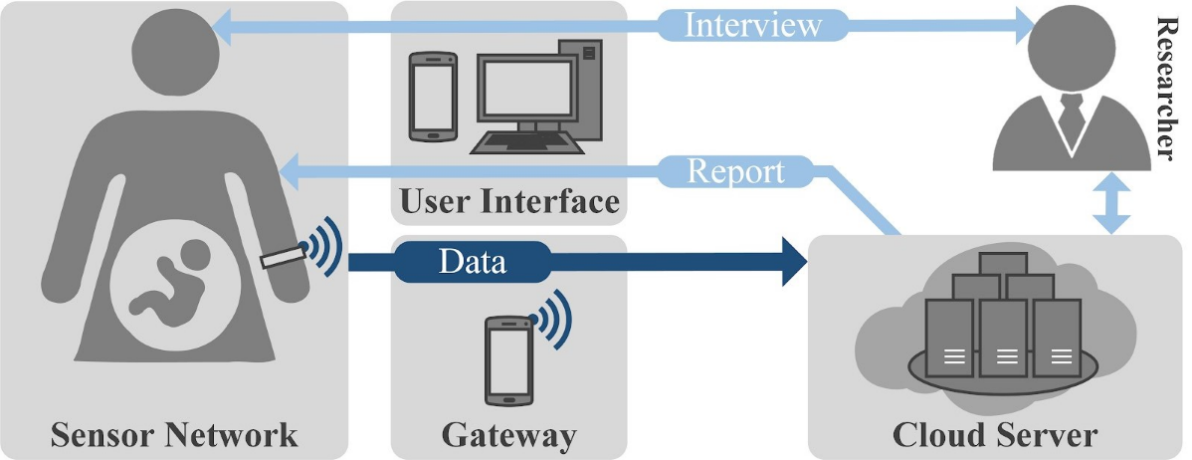
An Internet of Things–based system for remote maternity monitoring.

An IoT-based system was implemented to provide remote health monitoring throughout pregnancy and postpartum. The monitoring system leverages an amalgamation of different sensing, communication, and computing resources to collect, transmit, and analyze data [5,21]. The architecture of this system is illustrated in Figure 2. First, a smart wristband was used to collect health data from the mother remotely. Second, the collected data were transmitted to cloud servers via a gateway device, which was a smartphone or a personal computer. Third, the cloud was responsible for storing the data and for performing the following data preprocessing and analysis methods.

Data collection rates were not fixed throughout monitoring, as the Garmin provided the parameters at different rates. Therefore, we first homogenized the heart rate values by interpolating or averaging them. We then selected a 15-minute interval between successive values. Moreover, step counts were obtained from the Garmin. We leveraged step count values to specify physical activity levels. Sleep duration per night was extracted from the sleep data provided by Garmin. To validate this data, we carried out a manual cross-check between the sleep data and other data, including hand movement and heart rate values. The sleep data were corrected or removed in cases of mismatch.

### Statistical Data Analysis

Physical activity (step count) and heart rate data were accepted as valid for analyses if the participant wore the device for at least 10 hours per day while awake [22,23] at least 4 days per week [24]. The only exception was the week of delivery, from which all awake data were included in the analyses because the delivery week is shorter in most cases (if woman deliverers at 40+1 gestational week, this week includes only 2 days). Sleep measurements were considered successful if there was no off-wrist time during the sleep period [25] and at least 4 sleep periods measured per week. Sleep duration was defined as the number of minutes scored as sleep by the Garmin algorithm.

To describe the women’s background information and monitored health parameters (step counts, sleep and awake minutes, heart rate, and MVPA minutes), means, SD, or CI and medians with ranges were used as continuous variables and counts with proportions for categorical variables. Daily step counts, sleep, and heart rate data from 13 weeks of gestation to 1 month postpartum were presented for the second and third trimesters and the postpartum period (averaging all accepted data during each period). The changes in weekly step counts and sleep minutes were assessed throughout pregnancy. These analyses were performed with a linear mixed model with repeated measures, including one within factor (time = gestational weeks). A compound symmetry covariance structure was used for time. This method allows women with missing values to be included in the analyses. The same method was used to study differences in average step count, sleep, and awake minutes between pregnancy trimesters. Changes over trimesters in MVPA minutes were analyzed using the Friedman test because the normality assumption was not met.

Spearman correlation testing was used to explore associations between mean daily step count by the second and third trimesters and the postpartum period as well as mean daily sleep minutes by the trimesters and postpartum period. MVPA minutes were compared to the recommendation for physical activity in healthy pregnant women: 150 minutes of moderate-intensity or 75 minutes of vigorous-intensity activity per week [26]. All tests were performed as two-sided tests with a significance level set at .05. The analyses were performed using SAS System, version 9.4 for Windows (SAS Institute).

## Results

### Participants

A total of 20 of 22 eligible pregnant women (refusal rate of 9%) were enrolled in the study. The mean gestational age at recruitment was 12 (SD 2) weeks of gestation. The background characteristics of the women are described in Table 1 and the perinatal outcomes are described in Table 2.

**Table 1:** Descriptive statistics for maternal background characteristics (N=20).

Characteristic		Participants
Age (years), mean (SD)		26 (5.0)
Gestational age at recruitment (week), mean (SD)		12 (2.1)
**Highest educational qualification, n (%)**		
	Primary education	4 (20)
	Secondary education	9 (45)
	College or university of applied sciences	4 (20)
	University	3 (15)
**Marital status, n (%)**		
	Married or living with a partner	17 (85)
	Single	3 (15)
**Employment status, n (%)**		
	Working	13 (65)
	Unemployed	2 (10)
	Student	5 (25)
Pre-pregnancy BMI (kg/m^2^), median (range)		24.4 (17.7–43.5)
Pre-pregnancy smoking, n (%)		7 (35)
Smoking during pregnancy, n (%)		5 (25)
Pre-pregnancy physical activity (almost daily), n (%)		12 (60)

**Table 2:** Descriptive statistics for perinatal outcomes (N=20).

Perinatal outcomes		Participants
Gestational age at delivery (week), mean (SD)		39.4 (2.6)
Gestational diabetes, n (%)		5 (25)
**Means of delivery, n (%)**		
	Vaginal	14 (70)
	Vacuum-assisted	4 (20)
	Emergency cesarean section	2 (10)
Birth weight (g), median (range)		3415 (1100-4445)

### Physical Activity and Sleep

The recordings yielded valid physical activity data for a median of 144 (range 13-188) days, which represented 75% of the 6-month data collection period during pregnancy. During the postpartum period, valid data were available for a median of 15 (range 0-25) days, representing 54% of the 1-month data collection period.

**Figure 3:**
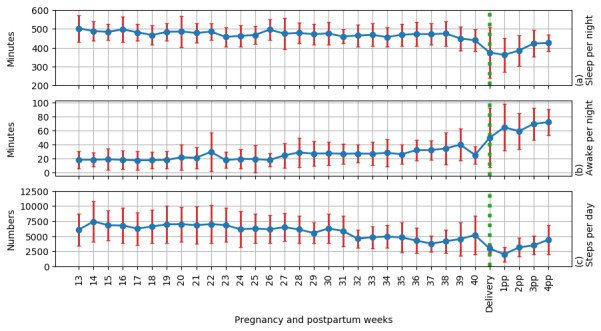
Mean and standard deviation of the sleep data and step counts per day by week during pregnancy and postpartum (n=2-19).

Physical activity decreased from the second trimester to the postpartum period (Figure 3). The mean daily steps during the second trimester were 6838 (95% CI 5866-7810) and decreased by a mean of 1793 (95% CI 1039-2548) steps per day in the third trimester (*P*<.001)*.* The daily steps further decreased by a mean of 1339 (95% CI 474-2205) steps in the postpartum period (*P*=.004). In weekly comparisons, the average daily step count was between 6000 and 7000 from 13-31 gestational weeks. After gestational week 32, the mean daily step count decreased to 5000 by 36 gestational weeks and further to approximately 4000 steps/day after that. The most significant decrease in physical activity occurred at 32 gestational weeks (*P*<.05 in most weekly comparisons) (Table 3).

Valid sleep data were available from a median of 137 days (range 0-184), representing 72% of data during pregnancy. During the postpartum period, a median of 16 days (range 0-27) of valid data (57%) was used in the analyses (Table 3). Sleep minutes decreased, and nightly awake minutes increased from the second trimester to the postpartum period (Figure 3). The participants slept a mean of 8 hours (95% CI 7.6-8.3 hours) during the second trimester and a mean of 20 minutes (95% CI –0.7 to 42 minutes) less per night in the third trimester (*P*=.06). The total night sleep time shortened an additional 1 hour (95% CI 39 minutes to 1.5 hours) in the postpartum period (*P*<.001)*.* In weekly comparisons, the average daily sleep minutes were between 450 and 500 from 13 to 38 gestational weeks. After gestational week 38, the mean daily sleep minutes decreased to 435 (*P*<.05 in most weekly comparisons; Table 3).

**Table 3:** Mean step counts and sleep minutes by trimesters and the postpartum period.

Variables		Second trimester (n=19)		Third trimester (n=17)		Postpartum period (n=12)		Overall differences between periods (*P* value)	
Valid physical activity days, n (%^a^)		76 (70)		51 (67)		12 (42)			
**Steps per day^b^**									
	Mean (CI)		6838 (5866-7810)		5045 (4049-6041)		3705 (2635-4776)		<.001
	Pairwise differences between periods (*P* value)		<.001^c^		.004^d^		<.001^e^		
Valid sleep days, n (%^a^)		69 (63)		48 (63)		12 (41)			
**Sleep (min/night)^b^**									
	Mean (CI)		477 (433-501)		457 (433-481)		393 (367-420)		<.001
	Pairwise differences between periods (*P* value)		.06^c^		<.001^d^		<.001^e^		
**Awake (min/night)^b^**									
	Mean (CI)		21 (15-27)		32 (25-40)		67 (52-81)		<.001
	Pairwise differences between periods (*P* value)		.02^c^		<.001^d^		<.001^e^		

^a^Calculated days during the follow-up period.

^b^Measured by Garmin Vivosmart.

^c^Second trimester versus third trimester.

^d^Third trimester versus postpartum period.

^e^Second trimester versus postpartum period.

### Heart Rate and the Intensity of Physical Activity

**Figure 4:**
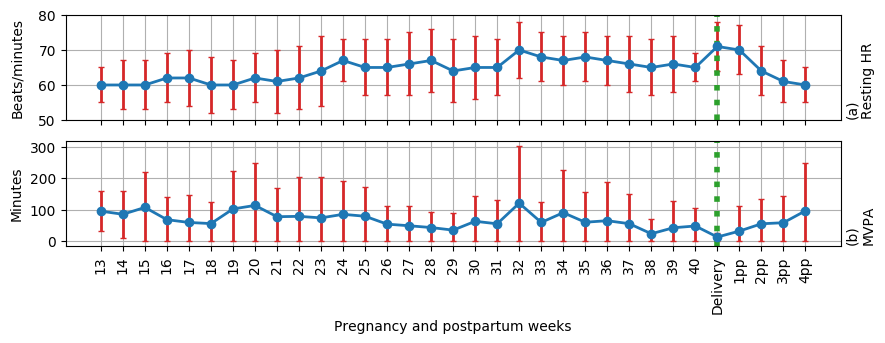
Resting heart rate values and moderate-to-vigorous physical activity minutes per week during pregnancy and postpartum (n=3-19). HR: heart rate; MVPA: moderate-to-vigorous physical activity.

The resting heart rate increased progressively by 17% from 60 bpm (SD 5) at 13 gestational weeks to 70 bpm (SD 8) at 32 gestational weeks and remained at that level until delivery. The resting heart rate decreased to the early pregnancy level by 4 weeks postpartum (Figure 4).

The intensity of physical activity decreased from the second trimester to the postpartum period. A median of weekly MVPA minutes during the second trimester was 46 (range 0-288) and decreased by a mean of 14 minutes (95% CI –3 to 32 minutes) per week in the third trimester. After delivery, the MVPA minutes further decreased to a median of 15 (range 0-188) minutes per week, but the change was not significant (*P*=.08; Figure 4). Comparing the MVPA minutes with the general recommendation of physical activity for pregnant women, only 47%, 24%, and 25% of the participants achieved the recommended activity level in at least 1 week in the second and third trimesters and the postpartum period, respectively (Table 4).

**Table 4:** Median MVPA minutes by trimester and the postpartum period.

Variables	Second trimester (n=19)	Third trimester (n=17)	Postpartum period (n=12)	Overall difference between periods (*P* value)
MVPA^a^ (min/week)^b^, median (range)	46 (0-288)	27 (0-279)	15 (0-188)	.08
Participants meeting the recommended MVPA^c^, n/N (%)	9/19 (47)	4/17 (24)	3/12 (25)	N/A^d^
Weeks of 150 MVPA minutes, n/N^e^ (%^c^)	43/239 (18 )	19/154 (12)	7/40 (18)	N/A

^a^MVPA: Moderate-to-vigorous physical activity.

^b^Measured by Garmin Vivosmart.

^c^Moderate-to-vigorous physical activity at least in 1 week during follow-up.

^d^N/A: not applicable

^e^Calculated weeks during the follow-up period.

## Discussion

### Principal Findings

To our knowledge, this is the first continuous, long-term follow-up study measuring physical activity, sleep, and HR during pregnancy and the postpartum period. The results of this study confirmed that this IoT-based system was feasible to monitor health parameters during pregnancy when we collected a large amount of valid physical activity (75% of the data collection period) and sleep (72%) data. After delivery, the amount of data obtained was not representative. Overall physical activity, measured objectively with step counts, was low during pregnancy and postpartum. Sleep time decreased during pregnancy and after delivery, and heart rate increased due to hemodynamic changes. These expected results support the preliminary reliability of a smart wristband with IoT-technology. However, more research is needed to validate the continuous monitoring of pregnant and postpartum women.

### Evaluation of the Health Parameters: Physical Activity, Sleep, and Heart Rate

Physical activity (daily steps) decreased during pregnancy and were quite low in the third trimester and especially during the first month after the delivery. Previous studies using intermittent and subjective measurements also showed that physical activity decreases as the pregnancy progresses [27,28], and one previous study using continuous monitoring also found that step counts decrease in inactive pregnant women as pregnancy proceeded [29]. According to a previous review [15], the smart wristband itself might increase participant physical activity. However, the effects of physical activity interventions are often short term. Thus we estimated that this was not relevant in our study due to the long follow-up period. After the delivery, it was not expected that the women’s physical activity would have returned to the early pregnancy level during the first month. In addition to the recovery from delivery, the transition to first-time parenthood takes time. A smart wristband could be used to illustrate the level of physical activity to a pregnant woman herself and, by implication, support her in changing her lifestyle to a more active one. Furthermore, physical activity improves sleep quality during the postpartum period [12,30], and well-rested women are most likely to be physically active; thus, both elements of health should be equally supported.

Our study confirmed previous findings on declining sleep quality and more frequent nocturnal awakenings. In line with the previous study [31], we found sleep quality decline starting from the ﬁrst trimester. In addition, both the number and duration of nocturnal awakenings seemed to increase as pregnancy proceeded [30] and in the postpartum period [12]. However, the sleep time remained rather high throughout pregnancy and after delivery. Pregnant women’s abnormal sleep duration is associated with several maternal problems, such as hypertensive disorders [32], increased body mass index after delivery [33], and depression and anxiety [34]. Moreover, extreme sleep duration during pregnancy has been associated with gestational diabetes [35]. Although the sleep duration of the study participants was mostly sufficient according to the present recommendations, the awake minutes during sleep periods increased during pregnancy. The nocturnal awake minutes reduced sleep quality and probably compromised sleep efficiency. The data on sleep quality in pregnancy are limited; thus, the present study adds significantly to the present knowledge of this subject. Smart wristbands provide many opportunities to measure and support the sleep hygiene of pregnant women in maternity care.

The resting heart rate increased until 32 gestational weeks, a normal hemodynamic change during singleton pregnancy [36]; thus, it appears that a smart wristband is an appropriate tool for measuring heart rate during pregnancy. In the future, heart rate and heart-rate variability could be utilized to study the level of stress or the recovery from physical or mental exertions. Maternal stress is associated with some pregnancy complications [37], and identifying the increase in stress could help with finding suitable interventions to support the pregnant woman. The intensity of the physical activity levels in this study was low, as expected, and decreased considerably during the last month of pregnancy. A comparison with the global physical activity recommendation for pregnant women showed that over half of the participants did not reach the recommended 150 minutes of moderate or vigorous physical activity per week during the second or third trimester.

### Limitations

The study includes distinct limitations due to the feasibility design. The participants were recruited as a convenience sample; however, the sample consisted of a diverse group of primiparous women. Due to the small sample size, the results from this study may not be generalizable. Furthermore, the small sample size did not allow us to perform statistical analyses between outcome predictors and maternal background characteristics or perinatal outcome variables. Although Garmin Vivosmart has been shown to provide a valid measure of step count [18,19] and total sleep time compared with a sleep diary in a healthy adult population [38], it is notable that the device has not been validated in pregnant women. A sleep diary would have strengthened our results. However, due to the extended data collection period, we made the decision not to use such a method. The intensity of physical activity was estimated based on heart rate elevation. This approach is not unambiguous in pregnant women, because even if the resting heart rate increases, the maximal heart rate decreases during pregnancy, resulting in a smaller heart rate reserve.

Furthermore, the specificity of intensity measures is only moderate as the device may underestimate the intensity at higher rates [19]. Therefore, moderate and vigorous activity minutes may be biased. Missing data due to the participants not wearing the smart wristband or not remembering to synchronize the device might have affected our results. The data collection period was, however, very long, and only valid weeks were included in the analysis. The data during pregnancy were representative and covered the long follow-up period well. After delivery, however, other methods for monitoring should be considered. In addition, the smart wristband used in this study was found to be a feasible tool for continuous monitoring during pregnancy [39].

### Conclusions

The smart wristband with the IoT solution seems to be a feasible system to collect representative data on continuous variables such as physical activity, sleep, and heart rate throughout pregnancy. However, more research is needed, and other methods for monitoring after delivery should be considered. Continuous monitoring provides personalized information and thus may help target and tailor pregnancy follow-up. Remote monitoring provides vast opportunities to observe health-related parameters in pregnancy and thus detect pathological signs at an early stage and partly replace traditional appointments. Future studies on predicting complications or support of women with high-risk pregnancies are needed.

## Abbreviations

GDM: gestational diabetes mellitus
IoT: Internet of Things
MVPA: moderate-to-vigorous physical activity
